# Children with palliative care needs in Papua New Guinea, and perspectives from their parents and health care workers: a qualitative study

**DOI:** 10.1186/s12904-023-01177-6

**Published:** 2023-06-08

**Authors:** Villa Watch, Gwenda Anga, Cornelia Kilalang, Francis Pulsan, John D Vince, Trevor Duke

**Affiliations:** 1 Paediatrician, Eastern Highlands Provincial Hospital, Goroka, Papua New Guinea; 2 Paediatrician – Oncology, Paediatric Clinical Coordinator, Port Moresby General Hospital, Port Moresby, Papua New Guinea; 3 Chief Paediatrician, National Department of Health, Port Moresby General Hospital, Port Moresby, Papua New Guinea; 4 Paediatrician and Senior Lecturer, School of Medicine and Health Sciences, University of Papua New Guinea, Port Moresby, Papua New Guinea; 5 Deputy Dean and Head of Research and Post-graduate studies School of Medicine and Health Sciences, University of Papua New Guinea, Port Moresby, Papua, New Guinea; 6 School of Medicine and Health Sciences, University of Papua New Guinea, Port Moresby, Papua New Guinea; 7 Department of Paediatrics and Intensive Care, Royal Children’s Hospital, Melbourne, Australia

**Keywords:** Paediatrics, Palliative care, Melanesia, Oceania

## Abstract

**Introduction:**

The World Health Organisation defines paediatric palliative care (PPC) as caring for the child’s body, mind, and spirit, and giving support to the family. In life-limiting conditions it is important that palliative support can be provided even when curative attempts are being utilised. In Papua New Guinea, as in other low- and middle-income countries there is a lack of services and training on PPC. This study aims to describe the characteristics of children with palliative care needs and to assess the perspectives of their parents and health care workers.

**Methods:**

A descriptive qualitative study was carried out over 5 months in 2022 at the Port Moresby General Hospital children’s wards. Clinical information was gathered from the admission charts of children with life threatening and life limiting conditions and a recorded interview was carried out with the children’s parents. A focus group interview with 10 experienced nurses caring for these children was video recorded. The recorded interviews were subjected to thematic analysis.

**Results:**

Twenty children and their parents were included in this study. Nine had a cancer diagnosis and 11 had a chronic progressive condition. The common clinical characteristics of children with palliative care needs were pain (n = 9) and shortness of breath (n = 9), and most children had more than one symptom. Several themes were identified in the parent interviews. Most parents could not name their child’s diagnosis, but they were able to correctly describe their child’s condition using their own terms. Most parents felt involved in their child’s management and were satisfied with the care provided. Parents were psychologically affected by their child’s situation but were hopeful that God and the medicines would heal their child. Ten nurses were involved in a focus-group interview. Most nurses’ understanding of palliative care was from experience not from formal training, but most felt confident in identifying the physical, emotional, and spiritual needs of the children. Understanding of analgesia was limited, as was the availability of appropriate medications included in the WHO Analgesic Ladder.

**Conclusion:**

There is a need for a systematic approach to palliative care in Papua New Guinea. Palliative care can be integrated into an overall approach to quality of paediatric care. It is relevant to a broad section of children with severe chronic or malignant conditions and can be carried out with limited resources. It does require some resources, further training and education, and increased provision of basic drugs for symptom control.

**Supplementary Information:**

The online version contains supplementary material available at 10.1186/s12904-023-01177-6.

## Introduction

According to the World Health Organisation paediatric palliative care (PPC), involves the child’s body, mind, and spirit, and giving support to the family. It is important that the care begins when illness is diagnosed and continues regardless of whether a child also receives curative treatment. Health care providers must attend to a child’s physical, psychological, and social distress. To achieve this often requires a broad multidisciplinary approach that includes the family and available community resources. Despite the challenges, children can receive good PPC even if resources are limited [1–4].

In 2014, the World Health Assembly (WHA 17.19) passed a resolution on “Strengthening Palliative Care for Children”, emphasizing that the accessibility of palliative care for children is an ethical responsibility of health systems [5]. However, PPC has not been seen as a priority around the world despite the obvious needs. In 2018 WHO reported up to 21 million children needed palliative care and about 2.5 million children died with health-related suffering. 98% of such deaths are in the low- and middle-income countries (LMIC) [1]. In Papua New Guinea and other developing nations in the Oceania region, there have been no studies on paediatric palliative care needs or perceptions.

We aimed to describe the characteristics of children with palliative care needs who were admitted to the paediatric ward of Port Moresby General Hospital; to assess their parents’ perspectives of palliative care; and to assess health care workers’ ability to provide palliative care to a child with such needs.

## Methods

### Study design and setting

This was a descriptive qualitative study, carried out in the paediatric wards of the Port Moresby General Hospital from March to July 2022. As the only tertiary-level hospital in PNG, PMGH is where children from all other provinces are referred when further specialist management is needed; this includes the investigation and management of many life limiting conditions for which children may at some stage have palliative care needs.

### Identification of patients as participants in the study

Children who were included in the study were drawn from four categories of life-threatening conditions (Table 1).

**Table 1:** Categories of life-threatening illness

Category 1	Category 2	Category 3	Category 4
Children with life-threatening conditions for which curative treatment may be feasible but can fail Cancers Complex congenital heart disease	Children with conditions in which there may be long phases of intensive treatment aimed at prolonging life, but premature death is still possible HIV Chronic lung disease	Children with progressive conditions without curative treatment Thalassemia, Neurodegenerative conditions, metabolic conditions, and neuromuscular conditions	Children with conditions with severe neurological disability, which may deteriorate unpredictably, but are not considered progressive Cerebral palsy, extreme prematurity, hypoxic brain injury

Adapted from: *Practical guide to palliative care in paediatrics*. Children’s Health Queensland Hospital and Health Service 2014, and Downing et al. [6]

### Data collection and analysis

Twenty children and their parents were included in the study. Diagnoses, clinical characteristics, palliative care needs, and interventions were recorded from their medical records and interviews.

Parents and guardians were interviewed by the first author (VW), using a questionnaire with semi-structured open questions (online Appendix). The participant parents were selected based on their child’s illness, and their willingness to be interviewed, in a convenience sample. No parents declined to be interviewed, although sometimes, guardians or parents were not in bed, so the interviewer would move on to other patients. The interviewer would sit with the parents or guardians and explain the aims of the interviews and they were mostly curious and willing to participate. The interviews were recorded on a recording application on an Android phone, and transcribed verbatim, and translated if in *Tok Pisin* (the *lingua franca* of PNG) or a local language (PNG has over 400 languages).

A focus group discussion was carried out with 10 health care workers (nurses) who were involved in providing palliative care to children, and willing to be interviewed. The focus group discussion was video recorded.

The interviewer was a female, a paediatric registrar (medical officer) at the time of the study, studying for a post-graduate masters specialist qualification in Child Health. She is now a paediatrician. Her relationship with the parent participants and the health care workers was based on her role as a paediatric registrar. Although not having received formal training in qualitative research, the interviewer had many years experienced in caring for children with life-limiting illnesses.

The interviews with parents or guardians generally lasted 45–60 min. Parent or guardian interviews and the health care workers focus-group discussion were transcribed. Comments not related to the study topic were omitted. The responses were thematically arranged, the content analysed, and responses that represented the range of views on a particular theme were grouped and noted. Thematic analysis as published by Braun and Clarke [7] was used to analyse the data with the goal of identifying themes and patterns that are interesting or important and involves a five-step framework (Table 2). Thematic analysis is a form of analysis that looks across all data to identify the common issues that recur and identifies the main themes that summarize all the views expressed.

**Table 2:** Qualitative methods

Phase	Examples of procedure for each step
Become familiar with the data	Transcribe data; read and re-read; devise and note initial codes
Search for the themes	Organise codes into potential content themes, gather all data relevant to each potential theme
Detailed review of the themes and responses	Check if the themes work in relation to the coded extracts and the entire dataset; select appropriate extracts and verbatim responses to reflect the range of opinions expressed.
Define the themes	Ongoing analysis to refine the specifics of each theme; generate clear names for each theme and ensure the range of responses are expressed.
Produce the report	Final analysis; discuss the themes and their meaning; relate back to research question or literature; write manuscript

## Results

Of the 20 children, 11 were male. Ages were < 1 year 3 (15%), 1–5 years 6 (30%), 6–10 years 4 (20%), 11–15 years 6 (30%), and 16–20 years 1 (5%). Thirteen came from Port Moresby and 7 had been referred from outer provinces that required air travel.

Nine children had cancer (5 retinoblastoma, 2 acute lymphoblastic leukaemia, 1 neuroblastoma and 1 germ cell tumour). Eleven had non-cancer diagnoses (4 post tuberculosis bronchiectasis and pulmonary hypertension including one with HIV and 2 with multi-drug resistant tuberculosis, 2 complex congenital heart disease, 2 post tuberculosis severe cerebral palsy, and 1 each of severe thalassaemia and hemosiderosis, multiple congenital anomalies and severe Guillain Barre syndrome).

Most children were in category 1 of life-threatening diagnoses: an illness where curative treatment may be feasible but may fail, including cancer. 20% had category 2, an illness that may involve long phases of treatment aimed at prolonging life, but premature death is still possible, such as HIV and bronchiectasis. 10% and 15% were categories 3 and 4 respectively: progressive illnesses with no curative treatment and illnesses like cerebral palsy, conditions that may deteriorate unpredictably, but are not generally progressive.

The main significant symptoms experienced by these patients at the time of interviews included pain (9), dyspnoea or respiratory depression (9), visual loss (5), bleeding and anaemia (5), feeding difficulties (3), severe motor dysfunction and mobility impairment (3), and fever (2). Treatments provided that have a palliative role were analgesics (9 received paracetamol, only one received morphine and pethidine), oxygen therapy, physiotherapy, nasogastric feeding, and blood transfusions.

The caregivers interviewed included 8 fathers, 7 mothers, 3 grandmothers, and one each sister, auntie, and uncle. The themes explored in the interviews included: the parents understanding of their child’s illness, the parents’ perception of the child’s symptoms, the emotional burden the child’s illness had on the family, and to what extent the parents felt supported by health care staff.

Three parents knew their child’s diagnosis, however 16 could describe it in their own descriptive terms but did not know the name, and 1 was unsure.

Most parents/guardians were able to understand their child’s illness even without correctly naming the diagnosis and with that understanding, most were able to know the respective palliative care needs of their child and help accordingly at home or in the hospital.

“Heart disease problem, em gat hol lo heart” ***(It is a heart problem, there is a hole in the heart)***

“Problem lo lung, pressure high lo lungs blo em so wokim em sotwin” ***(It is a problem of the lungs, the pressure is high in her lungs, so she has shortness of breath)***

“Mi no clear lo name blo sik but em fungus” ***(I do not know the name of the illness, but she is sick with a fungus)***

Parents or guardian had perceptions of the child’s palliative care needs and described it in their own descriptive language (Table 3):

**Table 3:** Parents perceptions of troubling symptoms and palliative care needs

Description from parents or guardians	Symptom or palliative care need	Number (%)
- “Em kisim taim lo pain” ***(He has terrible pain)*** - “Pain lo eye” ***(Pain in the eye)*** - “Headache”	Pain	6 (24%)
- “Em stap lo oxygen, em nonap survive em yet” ***(She is on oxygen and cannot survive on her own)*** - “Kus sa wokim now, em sa sotwin hariap” ***(When she develops cough, she is quick to develop shortness of breath)***	Dyspnoea	7 (28%)
- “Lek han paralyse, eye blind, hard lo sleep, em pilim pain, hard lo toktok, hard lo kisim kaikai lo maus so mipla putim lo tube” ***(She is not able to move her legs and hands, her eyes are blind, she has difficulty sleeping, she feels pain, she cannot talk, she has difficulty eating orally so we are feeding her through a tube)*** - “Em no toktok, em no kaikai lo maus so ol putim mipla lo tube na em sa kaikai. Sampla taim, traim feedim em lo maus” ***(She cannot talk and cannot eat orally so they put in a tube that helps her eat. Sometimes we try to feed her through her mouth)***	Cerebral palsy and feeding problems	2 (2%)
- “Eye solap na blackpla eye blo em go white. Eye bagarap” ***(He had swollen eyes and the black part of the eye turned white. His eyes are damaged)*** - “Eye blo em growth ya” ***(There is a growth in his eye)***	Visual loss	7 (28%)
- “Bleeding gums, platelet deficeiency, iron deficiency, cannot walk because of shortness of breath” - “Em i sotwin because em gat enlarged spleen, heart blo em ino wok normal so em sotwin” ***(He has shortness of breath because he has enlarged spleen; his heart is not working normally)***	Recurrent anaemia	2 (2%)

Parents and guardians expressed strong feelings about their child’s predicament (Table 4): 57% were anxious, sad and / or depressed. 14% of parents and guardians thought it was their fault while the other 14% of parents were at peace. When asked whether we had given them an opportunity to express how they felt: 50% of parents and guardians said, “no”; 45% said, “yes”; and one said, “they do not care how they felt”.

**Table 4:** Emotions expressed by parents regarding their child’s situation

Description from parents or guardians	Emotion	Number (%)
- “me worry”, “pilim hevi”, “upset”, “bagarap”, “tingting planti”, “mi cry”, “not happy”, “mind na lewa blo me hangamap” ***(I am worried; I feel burdened; I am upset; I feel terrible; I am anxious; I cry; I am not happy; My mind and heart are hanging)*** - “mi lukautim em go na me die now, em me worry lo disla” **(*I am caring for her now but I am worried about her care after I die)***	Sad, anxious or depressed	8 (57%)
- “em ba orait, me wanbel” ***(He will be okay, I am at peace)*** - “it’s unexpected but we accept it”	At peace with the diagnosis	2 (14%)
- “ mi fret” ***(I am scared)***	Scared	1 (7%)
- “em mas wrong blo mipla ol parents” ***(It must be our fault as parents***, ***we must have done something wrong)*** - “maybe mama blo em no kisim banis sut” ***(Maybe her mother was not vaccinated)***	Guilt and blame	2 (14%)
- “mi faul. How em kisim disla sik?” ***(I am confused as to how he got this illness)***	Confused	1 (7%)

Most parents (81%) were given the chance to talk about their child’s plan of management while 18% of parents were not happy, stating that they are still in the dark and that doctors talk to themselves most times during wards rounds and do not explain their child’s management plans in detail.

“Ol puttim oxygen tasol na me sindaun stap. Mi stap lo tutak yet” ***(They put him on oxygen and that is why I am sitting here. I am in the dark)***

“Lo ward rounds, planti taim ol dokta sa toktok lo ol yet. Ol no sa explain gut lo sik or marasin ol givim lo pikinini. Ol sa ting, mipla no sa understandim ol sapos ol explain lo mipla, ol ba tokim antap antap tasol” ***(During ward rounds, most times, doctors tend to talk amongst themselves. They do not explain well regarding my child’s illness or the medicines they are giving to my child. They think that we do not understand them if they explain to us. They explain superficially or briefly)***

63% of parents and guardians were satisfied regarding their child’s management praising the nursing team for being up to date with their child’s medication and other needs in the ward. 27% were not satisfied, one parent stating that she is still in the dark in terms of her child’s management.

“Em no pilim pain more” ***(He is no longer in pain)***

Nurses always up to date with meds, hot water, milk, they are on time

“Mi ting ol gim marasin ba orait liklik” ***(I thought that after the medicines were given, my child would be a little bit better)***

Not really good

I’m still in darkness

“Mipla go kam, go kam, mi lukluk tasol. Em orait or?” ***(We are going back and forth, back and forth, I am just observing. Is he going to be alright?)***

Parents were asked if the medical team have discussed the possibility of a poor outcome for their child. This had not occurred to the parents’ recollection in two-thirds. Most parents were not told of the possibility of having a poor outcome for their child, that is, either having a “poor quality of life” or “the possibility of death”.

Many parents showed faith, religious and otherwise: 25% of parents believed that God would heal their child and 20% believed that the medicines would heal their child. Another 20% were unsure and anxious, while the same percentage of parents (20%) were just angry. 10% of parents and guardians accepted the poor prognosis.

### Health care workers perspective on paediatric palliative care

We explored nurses understanding of palliative care, their perceived ability to identify a child who needs palliative care, and their skills and resources for addressing palliative care needs for a sick child. The 10 nurses that were involved in the focus-group interview were very experienced, an average of 16 years of experience, however none had any specific training in palliative care.

These nursing officers were asked to explain what palliative care is in their understanding, the answers focused more on the care of chronically ill children with a poor prognosis, and optimising quality of life or “happiness” for parents and families.

Care of disabled, long-term patients

Supportive care of patients with poor prognosis, to prolong life span

Care of patient with no future

Poor prognosis patients, provide care to make them happy; we attend to child so parents must be happy.

Patients with no future, will die anytime; we provide care and spiritual needs and physical needs as well; if they are in pain, we give pain relief. We provide support to keep them going as long as they are with us in the ward.

Main focus and aim is to ensure parents are happy; and prepared to accept what is expected of patient.

The staff expressed that after learning that a child is now requiring palliative care the focus is more on dying (80%), while others expressed the need to focus on the provision of holistic care for the child in the time they have left (20%).

Patient will get treatment and stay, then die

Patient will live for some years and die

We treat them to the limit and after that, send home to stay and wait for their death

We as nurses must think of patient as a whole; their physical, emotional, social and spiritual wellbeing. Help them physically e.g., supportive treatment, and emotionally we must help them and that helps their mental wellbeing as well as their spiritual.

We think ‘This patient won’t make it’ in the next week or next month; we might not keep patient for long. We address patient’s needs pain relief; spiritual counselling; help parents at least accept what the child is going through and the fact that child may not make it in the end.

Nurses felt they had the ability to identify a child who needs palliative care and how to address these needs. Most of the staff assessed the severity of pain in children based on their experiences, rather than on any formal teaching.

From experience, the child cries. In bigger children, they can communicate so they call out. They are restless and do not sleep.

From experience, children who can talk, they express their pain by crying and by saying, “*I’m feeling pain*.

I normally assess them; and give first line analgesic like Panadol and if it doesn’t help then we get a doctor to order something stronger to relieve pain so parents will be happy.

We get doctors to put on pain medicine which is stronger than what is written on drug sheet, when pain gets severe.

If the parents come and demand that we give (analgesia)

When to give pethidine; I assess pain. Some chronic patients have been on pain killers for so long, they are kind of addicted, sometimes they are pretending so you must assess. If you reassure them and they quieten down then its psychological; sometimes parents get frustrated, so they demand, but don’t listen to parents; make your own assessment.

Doctors may commence patients, especially cancer patients on pethidine, morphine or codeine; so, when its regular, 6-hourly, we follow and give; if in between they are still in pain then we get doctors to order alternative pain killers. We give medicine at scheduled times but most times, they need spiritual counsellors to be around.

Pain can also be managed by non-pharmacological therapy, some listed by the health care workers include outings, video games, music video, music, watching TV, herbal treatment, lollies and spiritual counsellor or a chaplain.

Apart from pain, other symptoms that can be relieved by appropriate palliative care were listed as: urinary retention, excessive sweating, fever, itchiness, generalised body weakness, not walking or sitting, bleeding, pus discharge, swelling, depression and fast breathing.

Nurses felt that many of the children under palliative care may need spiritual help and this could be identified by observation, or older children may ask to read a Bible verse, or through their parents.

You will observe them sitting down and thinking a lot; walk in and out without any reason and stand out and gaze around

“Children themselves cannot talk; but parents are confused, worried.

As a Christian nurse, if you see a patient and your spirit is telling you to pray for them, you will have the feeling that the patient needs prayer.

From my experience, they ask, ‘*Can you read me this scripture?*’ so spiritual part of treatment is very helpful, especially in older children.

Nurses expressed an ability to identify a child who needs psychosocial help, again through their experience rather than formal teaching.

“If a child is not happy, they will cry; if husband and wife, they will argue a lot, maybe they need money, so we need to help them financially Sometimes they are (parents) fed up of just being in the ward waiting for treatment so they start giving excuses, e.g., “mama/papa blo me die.” ***(My father/mother died)***

“They ignore us when we go to give treatment. They give their back to us. They will say, “*Puttim marasin lo hap!”****(Put my medicines there!)***

They refuse when we try to cannulate, “*Yupla sutim me planti taim*.” ***(You pricked me too many times)***. It’s hard to tell them, “You are sick, you should let us help you.” So, I leave the medicines and go back to them later when they are ready.

To help children with psychosocial needs, the staff identified two interventions, and that is to involve the psychiatric team and also individually, try to make friends with the child in order to gain their trust.

“I try to play with them and make them forget about their pain. You must first be friendly with them and then they will accept you. If you are harsh on them, you will not get it done. At least, for a few minutes, find time to sit and talk with them. If you share something with them then he or she becomes my friend, when I’m on duty, they say: “*oh, friend blo me!”****(Oh, that’s my friend!)***. Especially, chronic patients, they know nurses and become friendly with whoever they’re comfortable with.”

Most nurses (84%) agreed that there is a need for a specialist paediatric palliative care team:

As general nurses, we give medicine and other nursing care, but we don’t know how they think and feel.

In a stressful workplace, it is a need.

In oncology, we have patients that stay for a very long time in the ward. We need to talk to them day by day, encouraging them. We need a Palliative care ward and have our staff trained so that we know what we are doing.

At the moment, we are caring for patients through our experiences or pre-training days. We are doing our best, but we need to train our nurses

## Discussion

In this study we explored the palliative care needs of a group of children with life-limiting illnesses in Papua New Guinea, the perceptions of their parents, and among a group of experienced nurses the understanding and perceived skills of providing palliative care. PNG is a traditional and diverse Melanesian society, and most reports of the concepts of palliative care have come from western and high-income countries in Europe, North America, or Australia, with some from Africa, [8, 9] but never from Pacific Island nations.

The perceptions of parents in this study reflect traditional thinking, the strong influence of religion in PNG society, an acknowledgement and acceptance of the limited resources in health and medicine, and the universal human feelings of parents and health care workers to do the best they can for their children and those they care for.

The diagnosis of children with palliative care needs were varied. Some had cancer, but common also were other chronic progressive or disabling conditions. Most diagnoses were grouped into Category 1 of life-threatening illness (55%), where curative treatment may be feasible but may fail. Category 2 made up 20%, Category 3 10% and Category 4 15%. The utility of such categorisation of life-threatening illness is questionable, especially in resource limited settings like PNG where treatment options and prognoses for complex conditions are not the same as in high-income settings.

There is a need for a more organised approach to palliative care in PNG, and to include it as part of an overall approach to improving the quality of paediatric care. Educating and training of health care workers is essential; none of the nurses involved in this study had received any formal training in palliative care and their practice was based largely on experience. More junior nurses with less experience are likely to have less capacity to provide palliative care. Education would help orientate health care workers to the broader needs of patients with palliative care, not just terminal or end of life care. It would also convey the knowledge that not all palliative care services need be hospital-based, that some families would want to care for their child at home, and that there are ways to achieve this even in less resourced settings, given that it is a human right.

The most feasible and useful type of education on PPC may be case discussions. The nurses in this study showed a willingness to discuss their experiences and feelings, and case discussions can be the basis for shared learning. This could include asking health care workers to reflect on the care they were able to provide, and to give education on analgesia and other palliative interventions.

Palliative care involves a multidisciplinary team of nurses, doctors, social workers, psychologist, physiotherapist, non-governmental organisations, and parents and families as partners. We only included nurses and parents in this study, but broadening the scope of further research into palliative care to involve resident doctors and other health professionals would be important.

Pain management is hampered in PNG, as in many resource-limited settings, by lack of training in analgesia, a lack of appropriate child-friendly medications, and often by misconceptions of the dangers of opioids in patients with life-limiting illnesses. WHO recommends the use of the Analgesic Ladder and an approach that has three main principles: *By the clock, By the mouth, By the ladder* [10]. Using the WHO Analgesic Ladder and WHO Essential Medicines is an affordable, safe, and efficient approach to chronic pain control in palliative care in low resource settings [11–13].

Hospital based palliative care programs are feasible in LMICs [8]. A study from a referral hospital in Bangladesh successfully implemented a hospital-based paediatric palliative care service specifically designed for a resource-limited setting. The study described the considerations and initiatives that were used, which included [14].

- Raising awareness and sensitisation on palliative care.
- Education and training.
- Implementing a paediatric palliative care service.
- Collecting data to provide a detailed picture of the palliative care needs of children and families.

Palliative care fits within the Child and Adolescent Health Redesign approach of the World Health Organization, and the PNG Child and Adolescent Health Policy and Plan 2021–2030, which emphasise holistic care for children and adolescents of all ages, and optimisation of quality of life [15, 16].

### Limitations of the study

The study had a small sample size, the questions are inherently subjective, and we did not use software to code the data. Rather we used manual analysis, thought and discussion by the primary author, and amongst all co-authors.

## Conclusions

Palliative care can be integrated into an overall approach to improving quality of paediatric care. It is relevant to a broad section of children with severe chronic or malignant conditions, it can sit aside attempts at curative treatment, and can be successfully implemented with the limited resources found in many LMICs. Palliative care requires training and education, and better provision of basic drugs for symptom control. The nurses involved in this study had learned many aspects of palliative care from experience of working with such children, but a more systematic approach is needed.

## Electronic supplementary material

Below is the link to the electronic supplementary material.

Supplementary Material 1

Supplementary Material 2

## Data Availability

The dataset generated and analysed for the current study are not publicly available, due to reasons of patient confidentiality, but will be available from the corresponding author on reasonable request, in a fully de-identified manner.
