# Supplementary material for: Children with palliative care needs in Papua New Guinea, and perspectives from their parents and health care workers: a qualitative study
Source: BMC Palliat Care. 2023 Jun 8;22:68. doi: 10.1186/s12904-023-01177-6 (PMC10249208; doi:10.1186/s12904-023-01177-6)
Supplement: Supplementary file 1 — Supplementary Material 1 [file 12904_2023_1177_MOESM1_ESM.pdf]

Characteristics of Children in need of Palliative care with perspectives from parents and health care workers.

This is a study that is carried out as part of my Masters in Paediatric Program at the SMHS, UPNG, 2022.

The main aim of this study is to describe how Palliative care is integrated into the standard curative management of children who are admitted with life threatening and life limiting illnesses. I hope to identify the main palliative care needs and how we have addressed the various needs. The perspective of parents and healthcare workers will further help us understand how we can improve our services in terms of Palliative care.

I, Dr Villa Watch, declare that all information that is collected in this questionnaire is for study purposes only and will remain confidential.

If you agree to take part in this study, please sign: …………………… Witness: …………………………

DERMOGRAPHICS		
Name		
Age		
Sex		
Origin		
Address		
Diagnosis		Date of diagnosis:
Life limiting classification		Current Date:
Main PC needs identified:		How these needs were met in hospital
PHYSICAL<br><br>PSYCHOSOCIAL<br><br>SPIRITUAL		

PARENTS/GUARDIANS QUESTIONAIRES

1. May you tell me about your child's condition/diagnosis?

2. What is your child's main complaints/issues?

3. What else can we do to help your child?

4. Did we give you a chance to talk about your thoughts and feelings about your child's condition?

5. How do you feel about your child's condition?

6. Did we give you a chance to talk about your child's management plan?

7. How do you feel about how we a managing your child's condition?
8. Did we discuss the possibility of a poor outcome with you? E.g.: death or poor quality of life<br><br>9. How do you feel about the possibility of your child dying?<br><br>10. How do you feel about the possibility of your child having a poor quality of life?

STAFF QUESTIONAIRE (FOCUS GROUP INTERVIEWS)

Name
Senior Nursing Officers
1.What is Paediatric Palliative Care?<br><br>2. Have you had any training in Palliative Care?<br><br>3. What is your first instinct when you know that this patient is for Palliative care?
4. In terms of pain management in a child under Palliative care, how do you identify severity of pain?<br><br>5. When do you help a child under Paediatric Palliative Care who is in pain?<br><br>6. Can you identify atleast 3-5 other symptoms that can be relieved by appropriate Palliative Care?<br><br>7. How do you identify that a child needs spiritual help?

8. How do you help a child who needs spiritual help?

9. How do you identify a child with psychosocial issues?

10. How do you help a child with psychosocial issues?

11. What are some other non-pharmacological measures that can be used to reduce symptoms as part of palliative care?

12. How do you feel when you explain to families about the possibility of death of their child?

13. If a specialist team, trained in counselling and managing children with Palliative Care needs were here, do you think it will make it easier for you and the children's families? Yes, or no.

Explain your answer:
